# WRKY20 at work: coordinating sugar signaling and plant growth and metabolism

**DOI:** 10.1093/plphys/kiag289

**Published:** 2026-05-23

**Authors:** Nilesh D Gawande

**Affiliations:** Assistant Features Editor, Plant Physiology, American Society of Plant Biologists; Department of Biotechnology, School of Sciences, Woxsen University, Hyderabad, Telangana 502345, India

Higher plants produce sucrose during photosynthesis, which can be transported through the phloem to other nonphotosynthetic parts of the plant. Sucrose has multiple roles, serving as an energy source, a building block, and a signaling molecule essential for plant development, stress responses, and enhancing yield (Ruan et al. 2010). The key components in sugar signaling include Hexokinase (HXK1), Snf1-related kinase 1 (SnRK1), and Target of Rapamycin (TOR), which sense sugar and regulate plant growth and responses to nutrients and light (Li et al. 2024).

In plants, SnRK1 is activated under low-carbon or energy conditions and phosphorylates various target proteins, including transcription factors and metabolic enzymes, responsible for metabolic reprogramming (Peixoto and Baena-González 2022). SnRK1 is a heterotrimeric complex and comprises a catalytic kinase α subunit, KIN10 or KIN11, and regulatory subunits β and γ (Ramon et al. 2013). KIN10 can function independently of its other regulatory subunits (Ramon et al. 2019). KIN10 is involved in sugar signaling, and overexpression of *KIN10* results in hypersensitivity to glucose and ABA and increases leaf soluble sugar content (Jossier et al. 2009; Wang et al. 2019).

Transcription factors (TFs) bind to cis-regulatory elements in gene promoters and regulate gene expression related to various biological processes. Certain TFs play key roles in sugar metabolism and transport in plants. The WRKY TF is a large family that has a role in regulating stress tolerance, growth, and development in plants. These TFs also act as substrates for kinases and E3 ubiquitin ligases, regulating the trade-off between plant growth and defense (Yang et al. 2025a, 2025b). WRKY TFs consist of a WRKY domain of about 60 residues, with an N-terminal domain comprised of the WRKYGQK sequence and the C-terminal with a zinc finger structure. The WRKY TFs regulate target gene expression by binding to W-box cis-elements in gene promoters through their conserved WRKY domains and zinc-finger motifs (Rushton et al. 2012).

Ma et al. (2026) investigated the role of the *Arabidopsis thaliana* transcription factor WRKY20 in sugar signaling and the regulation of plant growth and development in a recent study published in *Plant Physiology*. The outline of this research is provided in Fig. 1. In their previous study, the authors identified *WRKY20* as a sugar-responsive gene (Chen et al. 2019). Using RT-qPCR, they found that *WRKY20* was induced in 2-wk-old *A. thaliana* Col-0 seedlings when treated with varying concentrations of exogenous sucrose under both light and dark conditions. To avoid biases caused by circadian rhythms or by sugars produced by photosynthesis, a similar experiment was performed in light using the electron transport inhibitor DCMU, which induces carbon starvation in seedlings in the light, as evidenced by the induction of starvation marker genes *DIN1* and *DIN6*. Conversely, these markers were suppressed with sucrose treatments, indicating that sucrose can alleviate the effects of carbon starvation in DCMU-treated seedlings. *WRKY20* was significantly induced by sucrose treatment compared to both the untreated control and DCMU-treated seedlings. The increase in *WRKY20* levels was twice as high in light as in the dark, suggesting that WRKY20 responds to both internal and external sugars.

**Figure 1:**
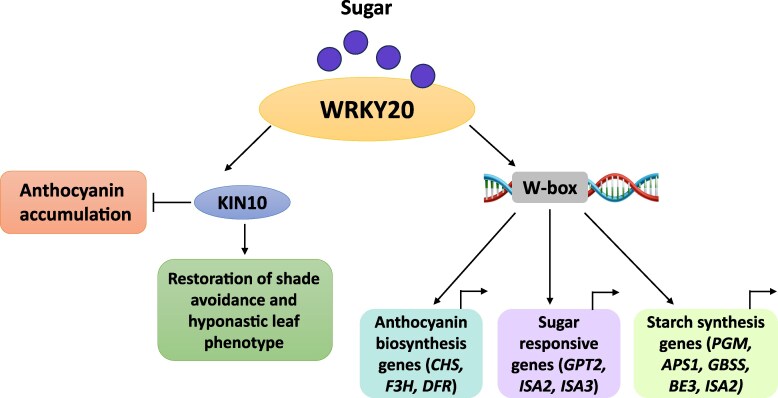
Proposed role of WRKY20 in sugar signaling and plant developmental regulation in *Arabidopsis thaliana*. The expression of transcription factor WRKY20 is induced by sugar. It binds to the W-box cis-elements present in the promoter region, further regulating the transcription of genes involved in anthocyanin biosynthesis (*CHS*, *F3H*, *DFR*), sugar responsiveness (*GPT2*, *ISA2*, *ISA3*), and starch synthesis (*PGM*, *APS1*, *GBSS*, *BE3*, *ISA2*). WRKY20 interacts with the SnRK1 kinase catalytic subunit KIN10, and overexpression of *KIN10* in *WRKY20-OE* plants suppresses anthocyanin accumulation and restores shade avoidance and hyponastic growth phenotypes, suggesting that WRKY20 acts upstream of KIN10.

*WRKY20* was strongly induced in the *A. thaliana* WT Ler ecotype in response to glucose treatment, whereas *WRKY20* expression levels were reduced in the glucose sensor HEXOKINASE1 (HXK1) mutant, *gin2-1*, suggesting that *HXK1* is involved in sugar-mediated induction of *WRKY20*. The analysis of the WRKY20 promoter region revealed several sugar-responsive cis-elements, including A-box, T-box, W-box, and 4 other responsive elements. The authors demonstrated sugar-induced expression of 3 fragments and the full-length promoter region of WRKY20 using transient expression of a luciferase (LUC) reporter in *Nicotiana benthamiana*, indicating the presence of sugar-responsive elements in the *WRKY20* promoter. However, the authors found that WRKY20 does not interact with the glucose sensor HXK1 in yeast 2-hybrid assays.

The authors further carried out the functional characterization of *WRKY20* using three independent CRISPR lines (*c20*, *c21*, *c23*). In addition, they also generated overexpression lines (*OE4*, *OE26*, *OE138*, and *OE169*) in Col-0 plants by introducing *35S* promoter-driven expression of *WRKY20*. In 6-d-old seedlings, they observed that high light treatment increased anthocyanin levels in overexpression lines, whereas it suppressed them in the *w20-cri* mutants. The increased light intensity enhances anthocyanin production by elevating sugar levels through improved photosynthesis and providing protection against photo-oxidative stress. The authors confirmed that higher light levels increased anthocyanin and sugar levels in the overexpression lines, whereas lower light levels decreased these levels in *w20-cri* mutants. These findings indicate that WRKY20 plays a role in sugar signaling and promotes anthocyanin production in response to higher light and sugar levels.

The authors further showed that anthocyanin biosynthesis genes like *CHALCONE SYNTHASE* (*CHS*), *FLAVANONE 3-HYDROXYLASE* (*F3H*), and *DIHYDROFLAVONOL REDUCTASE* (*DFR*) were significantly induced in overexpression lines, while the expression of these genes was repressed in *w20-cri* mutants. A similar trend was found for the sugar-induced marker genes *GPT2* (*Glucose-6-phosphate translocator2*), *ISA2* (*ISOAMYLASE2*), and *ISA3*. They further confirmed the binding of WRKY20 to the W-box regions of the promoters of these genes using a yeast 1-hybrid assay and the transcriptional activation of these genes by WRKY20 using a dual luciferase assay in *Nicotiana*. They further confirmed enrichment of promoter fragments of the *CHS*, *F3H*, and *DFR* genes in *WRKY20-FLAG-OE* lines by chromatin immunoprecipitation (ChIP) assays. These findings suggest that WRKY20 plays a direct role in modulating sugar-responsive and anthocyanin biosynthesis gene expression in sugar-treated seedlings.

Sugar has a crucial role in starch synthesis. The authors assessed leaf diurnal starch levels in *WRKY20-OE* plants and mutant lines using the iodine test on 2-wk-old plants grown under long or short-day conditions. Overexpression or mutant lines did not display significant changes in starch levels at T0. After 4 h of light exposure, *WRKY20-OE* lines showed higher starch content. Interestingly, starch levels were not reduced in mutant lines under short-day conditions, suggesting that WRKY20 positively regulates starch synthesis and may have a redundant role. Further, RT-qPCR analysis of *WRKY20-OE* lines displayed a higher expression for starch synthesis-related genes such as *PGM* (*PHOSPHOGLUCOMUTASE*), *APS1* (*ADP-glucose pyrophosphorylase small subunit 1*), *GBSS* (*Granule-bound starch synthase 1*), *BE3* (*Starch branching enzyme 3*), and *ISA2* (*Isoamylase 2*). CHIP-qPCR assays showed enrichment in these starch synthesis gene promoters. The authors confirmed the binding of WRKY20 to W-box cis-elements in the promoters of these genes using a yeast 1-hybrid assay and transcriptional activation in a luciferase reporter assay.

The authors further investigated the physiological role of WRKY20 across multiple stages of plant growth and development in *WRKY20-OE* and mutant lines. *WRKY20-OE* lines displayed larger seeds, wider cotyledons at the seedling stage, greater open angles of the third leaves, early flowering, an accelerated senescence phenotype, and reduced chlorophyll content compared to WT. These results suggest that WRKY20 regulates various morphological characters across different developmental stages in plants, although these effects may not be entirely direct, as altered sugar levels could also contribute to the observed phenotypes.

Toward identifying the molecular mechanism of WRKY20 regulation, the authors used yeast 2-hybrid assays to show that WRKY20 interacts with the catalytic subunit of SnRK1, KIN10. Overexpression of *KIN10* in *WRKY20-OE* suppressed anthocyanin accumulation and reversed the reduced shade-avoidance and hyponastic (upward leaf bending) phenotypes caused by *WRKY20* overexpression, suggesting that WRKY20 acts upstream of KIN10 to regulate anthocyanin synthesis and leaf growth responses.

In conclusion, this study highlights the significant role of WRKY20 in integrating sugar signals to regulate plant growth and development. Future research could focus on the functional characterization of the genes activated by WRKY20 and on investigating their roles in sugar signaling that influences plant growth. It would also be important to see if KIN10 phosphorylates WRKY20.

## Recent related articles in *Plant Physiology*:

1. Wang et al. (2025) investigated the role of plant microRNA 164 (miR164) and a sugarcane NAC transcription factor in sugar metabolism. They found that miR164 directly regulates Sc*NAC* expression through mRNA cleavage.
2. Yang et al. (2025a, 2025b) studied the roles of 67 Major Facilitator Superfamily transporters in sugar metabolism during peach fruit development using spatial metabolomics, transcriptomics, and functional studies.
3. Bi et al. (2025) explored the role of the ubiquitin E3 ligase CONSTITUTIVELY PHOTOMORPHOGENIC 1 (FvCOP1) in strawberry fruit quality and observed changes in sugars and anthocyanins with other components.

## Data Availability

Not Applicable.
